# Building Master Trainers to Facilitate Sexual Violence Prevention: A Pilot Study in Ghana

**DOI:** 10.5334/aogh.2747

**Published:** 2020-10-13

**Authors:** Caitlin M. Choi, Michelle L. Munro-Kramer, Lindsay M. Cannon, Ruth Owusu-Antwi, Angela D. Akorsu, Sarah D. Compton

**Affiliations:** 1 School of Nursing, University of Michigan Ann Arbor, MI, US; 2 Department of Sociology, University of Wisconsin-Madison, US; 3 Department of Psychiatry, Komfo Anokye Teaching Hospital, Kumasi, GH; 4 Department of Labour and Human Resource Studies, University of Cape Coast, Cape Coast, GH; 5 Department of Obstetrics and Gynecology, University of Michigan Ann Arbor, MI, US

## Abstract

**Background:**

Sexual violence is a widespread human rights violation that affects women and girls throughout the world, with particularly high rates among college-age youth. In the United States, many universities have developed primary prevention education programs to comply with federal mandates; however, these programs are limited in sub-Saharan Africa.

**Objectives:**

The purpose of this pilot study is to describe and evaluate the training of peer facilitators for a sexual violence prevention program at two universities in Ghana; the University of Cape Coast and Kwame Nkrumah University of Science and Technology.

**Methods:**

A three-day “master trainer” training was held focusing on sexual violence, sexual health, bias, healthy relationships, and facilitation skills. Participants completed pre- and post-test evaluations on knowledge, attitudes, and skills related to the topics and participants from the University of Cape Coast also participated in a focus group about bias and self-care.

**Findings:**

Participants (n = 23) at both universities demonstrated significant changes in the domains of: self-care knowledge, sexual violence knowledge, rape myth acceptance, and facilitation skills.

**Conclusions:**

This study provides early evidence about training methods for primary prevention programs aimed at students on university campuses in sub-Saharan Africa. Further research is needed on peer-facilitation, training, and primary prevention programs related to sexual violence for university students in sub-Saharan Africa.

## Introduction

In sub-Saharan Africa, nearly half of all women will experience some form of intimate partner violence or non-partner sexual violence in their lifetime [1]. Specifically, Ghanaian women experience various forms of violence ranging from female genital mutilation to intimate partner violence [2]. A 2004 study interviewed 50 Ghanaian women on their experiences and found that 70% experienced physical and/or sexual intimate partner violence. Violence against women is often normalized by perpetrators in relationships and many women may be socialized or forced to be compliant or submissive with sexual partners [2,3].

Recently, a 2017 study by Rominski et al. found that 25% of female students at the University of Cape Coast (UCC) in Ghana reported that their first sexual experience was forced or coerced [3]. Sexual coercion includes any strategy that threatens violence or harm to an individual if they do not engage in a sexual act. College-aged women are particularly vulnerable to experiencing sexual violence on university campuses due to a variety of sociocultural factors [4]. Ghanaian university students also report high levels of rape myth acceptance [5]. There is a need for culturally appropriate, peer-led sexual violence prevention programs on university campuses to address underlying causes of violence, such as knowledge about sexual violence, rape myth acceptance, and other social factors which normalize violence in relationships [6].

### Peer-Led Prevention Programs

Researchers suggest peer education provides both formal and informal learning processes, differing from a traditional model of learning with a teacher or figure of authority in charge [7]. Instead of having one person leading the teaching process, students are able to learn with each other and from each other [7]. In one peer education course among college fraternity members, students reported understanding sexual violence material in a contextual manner that helped them to have more emotional connections to what they were learning [8]. Other studies have found that peer-led programs focused on sexual violence are most effective at reducing rape myth acceptance [9], decreasing violence among men [10], and may have effects on bystander attitudes and efficacy [11] (although a meta-analysis by Jouriles et al., 2018 did not support this [12]). At the University of Michigan (UM), a peer-led primary prevention program for first-year students, aimed at reducing sexual violence, began in 2011. The ninety-minute program is an interactive training about healthy relationships, sex, and consent that has reached over 18,000 students in the first 5-year period it was offered between 2011–2015 [13]. A 2015 evaluation suggested the program resulted in significant changes in attitudes and knowledge around values-based decision-making, knowledge about how to ask for consent, and relationship communication skills [13,14]. Although some research suggests that students in Ghana experience similar rates of violence as students in the United States [3,4], there is a lack of peer-led primary prevention programs that have been proven to be effective in creating shifts in attitudes and behavior. One effective sexual violence prevention method is peer education, a strategy that has the potential to be effective in Ghana [15].

In response to this need, the research team used a systematic framework [16], to adapt the UM peer-delivered primary prevention program to the Ghanaian context [17]. As part of this adaptation, the research team conducted a training of master trainers who would be responsible for delivering the content of the primary prevention program and training others to deliver it. The training was conducted over two three-day periods, one at UCC in Cape Coast and one at Kwame Nkrumah University of Science and Technology (KNUST) in Kumasi, two cities in Ghana. The training-of-trainers (TOT) model will continue to be used to sustainably train peer facilitators. The TOT framework was modeled after successful TOT programs in low- and middle-income countries that have focused on maternal health [18], by utilizing experts on the topic to train a small cadre of master trainers at each new site to continue training qualified peer facilitators for the longevity of the program. This paper specifically reports on the creation and evaluation of the TOT model for sexual violence prevention in Ghana.

### Purpose

To our knowledge, the TOT model has not been used for prevention of sexual violence in universities in Ghana. This model of training brings a new perspective on prevention programs and has implications for how other programs are utilized in low- and middle-income countries. The purpose of this TOT curriculum, which uses a Facilitator Training Manual and interactive workshop, is to increase the knowledge, attitudes, and skills of master trainers at two universities in Ghana across a number of domains. The aims of this study are (1) to evaluate the effectiveness of a Facilitator Training Manual for the TOT model and (2) to gather pre- and post-test outcome data on perceptions of sexual violence, rape myth acceptance, gender equality, and facilitation skills among master trainers.

## Methods

To address the purpose of this study, we used a descriptive pilot design with pre- and post-test evaluations. The research team conducted TOT sessions twice: once at UCC in March 2017 and once at KNUST in July 2019. The team engaged current experienced student facilitators of the primary prevention program at the UM to help conduct the first training at UCC and master facilitators from UCC to conduct the training at KNUST, all under the supervision of the research investigators. This study received approval from the UM Institutional Review Board, the UCC Ethical Review Board, and the Committee on Human Research, Publication, and Ethics at KNUST.

### Procedures

A total of six female students (four undergraduate and two graduate) were selected from UM as peer facilitators to lead the TOT sessions over a three-day period at UCC in March 2017; each student led one 1.5-hour session. Research colleagues from UCC recruited the master trainers and facilitated the organization of the three-day training in Cape Coast. Master trainers were chosen by the University’s Centre for Gender Research, Advocacy, and Documentation (CEGRAD) and the Department of Population Health. Then, three master trainers from UCC who had extensive experience with peer facilitation and delivering the primary prevention workshop were selected to travel to KNUST in July 2019. Each master trainer led one 1.5-hour training session as well as collaborated on group training to present the primary prevention program, review the Facilitator Training Manual, and assist the KNUST facilitators in practicing the program. Master trainers at both sites received an invitation to participate in the research study based on the following inclusion criteria: (1) have experience studying the topics of sexual health and/or gender (UCC) or self-identified interest in peer-facilitation and sexual violence prevention (KNUST), (2) student or staff (UCC) or health science student (KNUST), (3) able to participate in the program for at least one year, and (4) willing to participate voluntarily in a three-day training session. The three-day training at both sites was evaluated using (1) qualitative methods (open-ended questions) and (2) pre- and post-test measures. Participants at UCC also participated in a semi-structured focus group discussion.

**Figure 1:**
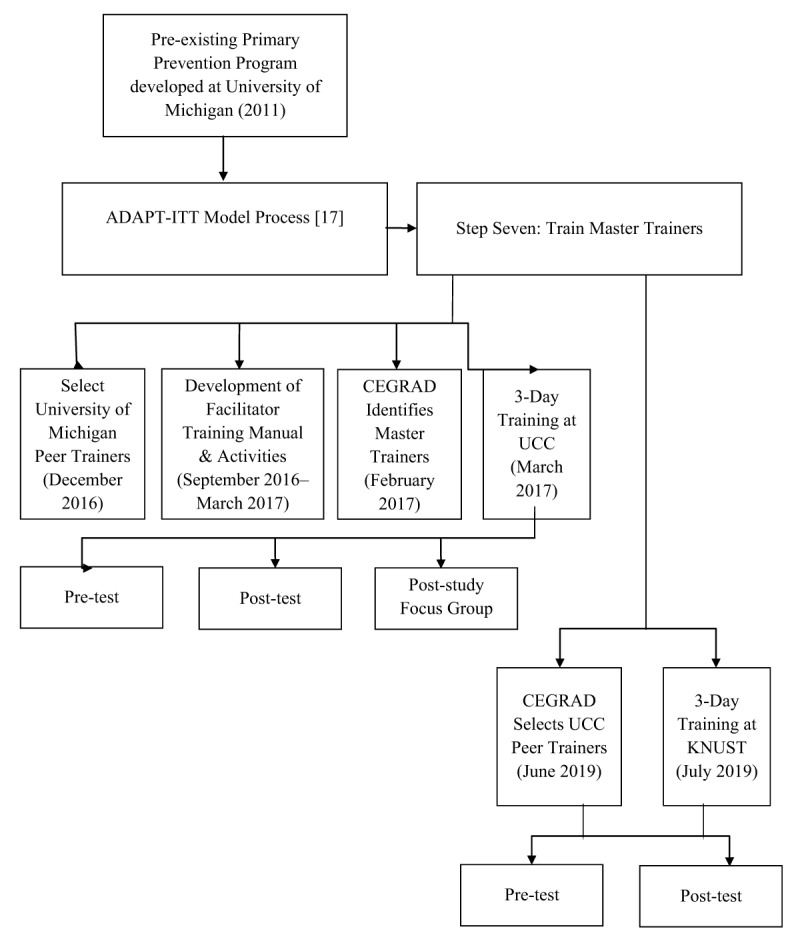
Study Procedures.

Participants (n = 23) at both sites were consented prior to any study activities (Figure 1). They reviewed the consent form and had the opportunity to ask questions. They were reminded that participation was voluntarily, that their participation or non-participation would not impact their schooling or job, and that they could stop participating at any time. The training and evaluations took place in private conference rooms on the UCC campus and the KNUST medical campus.

This training used a standardized Facilitator Training Manual that covered information related to sexual violence, sexual health, facilitation skills, identifying bias, and self-care (Table 1) and provided extensive information on the topics covered in each component of the program. A glossary of terms, common questions received by program facilitators, and tips on facilitation techniques were also included.

**Table 1:** Facilitator Training Manual Table of Contents.


1	Introduction
2	Language
3	Healthy Relationships
4	Gender Equality
5	Consent & Communication
6	Making Choices about Sex
7	Sexual Harassment*
8	Prevention of Sexual Harassment*
9	Self-Care
10	Facilitation Skill
11	Resources
12	Glossary
13	Appendices

* Sexual harassment is the umbrella term used to describe any unwanted sexual activity at these institutions (e.g., unwanted sexual comments, unwanted sexual contact, rape).

Sessions included an overview of sexual violence, foundational knowledge of sexual health, bias, healthy relationships, and, finally, two sessions focused on facilitation skills. The master trainer participants were equipped to navigate difficult questions through role-playing and skills-based activities. The final workshop focused on self-care, with the purpose of providing education and brainstorming ways the participants can incorporate self-care into their daily lives. In addition to these workshops, participants practiced the full-length program with partners as well as discussed the utility of the Facilitator Training Manual, and ways it could be improved. At UCC, master trainers also participated in a focus group discussion about bias, self-care, and overall perceptions of the TOT sessions on the last day.

### Evaluation Methods

Participants completed two surveys; one immediately before training began and one immediately following the conclusion of the training sessions. Pre-test surveys evaluated demographics, while both surveys evaluated knowledge, attitudes, and skills, as outlined below.

**Knowledge** of self-care, sexual health, and sexual violence (quantitative and qualitative) were measured using investigator-generated survey items (n = 19). Surveys assessed both trainee assessment of their own knowledge as well as objective knowledge. The 5-item investigator-created Self-Care Knowledge scale had possible scores ranging from 1 to 6, with higher scores representing greater knowledge of self-care practices. An example of a self-care knowledge question was, “I know how to define self-care.” Sexual Health Knowledge was measured by a 6-item investigator-created measure of sexual health, with 6 representing highest sexual health knowledge. Sample questions included objective items assessing knowledge about sexually transmitted infections and condoms. Sexual Violence Knowledge was assessed using five qualitative and two quantitative investigator-created items. Quantitative items were assessed on a scale from 1 to 5, with 5 representing greatest sexual violence knowledge. A quantitative example item was, “I feel that I am very knowledgeable about sexual violence.” Qualitative measures asked for definitions of key concepts related to sexual violence such as consent and coercion.

Healthy Relationship Knowledge was measured using an 8-item scale originally used to evaluate the primary prevention program in the United States [14]. Each item was scored between 1 and 3, where 3 represents greatest healthy relationship knowledge. Sample items included, “I know how to define what a healthy relationship looks like to me.”

**Attitudes** about rape myth acceptance and gender equality were measured using pre-existing scales that have been evaluated and adapted to the Ghanaian setting [17]. The modified Rape Myth Acceptance scale (n = 21) was used to measure rape myth acceptance [19]. The modified Rape Myth Acceptance scale is scored from 1 to 5, with higher scores representing greater rape myth rejection. Gender equality attitudes were measured with the Gender Equitable Men (GEM) Scale (n = 24), which measures attitudes toward gender norms [20]. Scale values ranged from 1 to 3, with higher scores indicating the most equitable gender attitudes.

**Skills** were evaluated with 12 investigator-generated items (quantitative and qualitative) on a Facilitator Skills scale, with higher scores indicating higher self-reported facilitation skills. The Facilitator Skills questions asked about creating a brave space for participants, engaging participants, and confidence in facilitation skills. The Peer Leader Survey (n = 8) was also used to measure characteristics of peer leaders [21], with a score of 6 representing highest self-reported peer leader skills. Due to a printing error at KNUST, pre-test response options were truncated and were standardized to the 6-point scale.

The focus group questions prompted discussion about bias, self-care, and overall perceptions of the facilitator training. The focus group included nine participants from the UCC training and lasted 34 minutes. Sample questions from the focus group were: (1) *Please describe what you liked about this facilitator training*, and (2) *After the workshop, how has your understanding of bias changed?*

### Data Analysis

The de-identified, quantitative data were analyzed using the Statistical Package for the Social Sciences (SPSS v. 25) to compare the pre- and post-test surveys. All *p*-values were set at 0.05. We used descriptive analyses, including paired *t*-tests, to evaluate changes in total scale scores from pre-test to post-test; *t*-tests are reported as absolute values. Quantitative outcomes were also computed for students by site (UCC and KNUST) to assess for changes at each location. Missing data were imputed using expectation-maximization imputation, an appropriate method based on the small percentage of missing data (1.0%) and small sample size [22].

Qualitative data from the post-study focus group discussion was transcribed verbatim. We used the constant comparative method to analyze the focus group discussion data to identify themes related to bias and overall perceptions of the training [23,24]. The qualitative analysis was conducted by three authors (CM, MMK, and LC). Each author read through the transcript in full to capture general thoughts, followed by in-depth reading of the transcript to identify and label general codes. Next, categories were developed for the codes. The transcripts and focus group notes were reviewed and categorized until each author believed that data saturation had occurred within their coding scheme [25]. After this, consensus was initially reached by the three authors that completed coding and then presented to all authors for consensus. Validity was maintained by utilizing an audit trail and validation with colleagues [26].

Quantitative and qualitative results were then triangulated to ensure completeness of data and to explain concepts (i.e., bias and self-care) that have not been well explored in Ghana [27]. The qualitative themes were interpreted and explained with the quantitative survey data in mind to ensure completeness of the data and interpretation.

## Results

### Descriptive Statistics

Demographics and sample characteristics are reported in Table 2. Pre-test data were missing for two UCC participants. As such, descriptive statistics of demographics and sample characteristics include data from all 23 participants, while *t*-tests are reported for only the 21 participants with complete data. Data from paired *t*-tests are reported in Table 3.

**Table 2:** Demographics and Sample Characteristics (n = 23).

Variable	N	Range	Mean ± SD

*Age*	23	21–33	24.4 ± 2.78
			**Percentage**

*Institution*			
University of Cape Coast (UCC)	10		43.5
Kwame Nkrumah University of Science and Technology (KNUST)	13		56.5
*Gender*	23		
Female	13		56.5
Male	10		43.5
*Role at the University*			
Undergraduate Student	1		4.3
Senior Medical Student	13		56.5
Graduate Student	7		30.5
National Service Participant	2		8.7
*Religion*			
Christian	21		91.3
Muslim	2		8.7
*Religious Attendance Frequency*			
More than weekly	15		65.2
Weekly	7		30.5
Less than monthly	1		4.3
*Ethnicity*			
Akan	13		56.5
Ewe	3		13.2
Ga/Adangbe	2		8.7
Nigerian	2		8.7
Mole-Dagbani	1		4.3
Chokosi	1		4.3
Voltarian	1		4.3

*Note*: These data represent the full study-sample of 23 master trainers. Two participants are excluded from the paired *t*-test analyses due to missing pre-test data.

**Table 3:** Paired t-test Comparisons of Pre-test and Post-test Scores (n = 21).

	Pre-Test		Post-Test		*t*-test

	M	SD	M	SD	

Self-Care Knowledge^1^	19.1	5.8	29.3	1.8	10.19***
Healthy Relationships Knowledge^2^	22.4	1.6	24.0	0.0	1.62***
Sexual Violence Knowledge^3^	7.5	1.6	9.4	1.5	1.95***
Sexual Health Knowledge^4^	4.9	0.9	4.8	0.8	–0.05
Modified Rape Myth Acceptance^5^	80.0	15.1	93.1	12.2	13.13***
Gender Equitable Men (GEM) Scale^6^	66.4	4.5	68.8	4.5	2.37***
Facilitation Skills^7^	21.3	5.9	32.5	4.6	11.19***
Peer Leader Skills^8^	36.3	6.8	41.5	9.6	5.17**

*Note*: M = Mean. SD = Standard Deviation. * *p* < 0.05, ** *p* < 0.01, *** *p* < 0.001. ^1^ Self-Care Knowledge scale (n = 5), scale range 5–30, higher scores representing greater knowledge of self-care practices. ^2^ Healthy Relationship Knowledge (n = 8), scale range 8–24, higher scores represent greater healthy relationship knowledge. ^3^ Sexual Violence Knowledge (n = 2), scale range 2–10, higher scores represent greater sexual violence knowledge. ^4^ Sexual Health Knowledge (n = 6), with 6 representing highest sexual health knowledge. ^5^ Modified Rape Myth Acceptance scale (n = 21), scale range 21–105, higher scores represent greater rape myth rejection. ^6^ Gender Equitable Men (GEM) Scale (n = 24), scale range 24–72, higher scores indicate more equitable gender attitudes. ^7^ Facilitator Skills scale (n = 7), scale range 5–35, higher scores indicate higher self-reported facilitation skills. ^8^ Peer Leader Survey (n = 8), scale range 8–48, higher scores indicate higher self-reported peer leader skills.

Ten participants from UCC (43.5%) and thirteen participants from KNUST (56.5%) participated in the training. Ten participants were male (43.5%) and thirteen participants were female (56.5%). Ages ranged from 21 to 33 years (M = 24.4, SD = 2.78). One undergraduate student (4.3%), thirteen senior medical students (56.5%), seven graduate students (30.4%), and two National Service Scheme participants (8.7%) took part in the training. National Service Scheme participants are graduates of accredited tertiary institutions completing an obligatory year of national service at a Ghanaian institution to which they are assigned.

### Summary of Themes

Qualitative data analysis resulted in four overarching themes that were consistent with the emphasis on attitudes, knowledge, and skills. The first theme, *knowledge*, included the sub-themes of self-care, healthy relationships, and sexual violence knowledge. These data are supported by quantitative and open-ended qualitative data from the Sexual Violence Knowledge questions as well as quantitative data from the Self-Care Knowledge and Healthy Relationship Knowledge scales. The second theme, *attitudes*, included the sub-themes of sexual violence and bias, and is supported by quantitative data from the GEM and modified Rape Myth Acceptance scales. The third theme, *learning styles*, included the subthemes of engagement, peer-to-peer learning, and training logistics. The final theme, *skills*, included the subtheme of facilitation techniques, and is supported by quantitative data from the Peer Leader Survey and Facilitator Skills scale.

#### Knowledge

During the focus group, participants expressed growth in knowledge in two areas: self-care and components of healthy relationships. Self-care emerged as a sub-theme since it was a new concept to participants and something they were interested in learning more about. Participants saw self-care as something they should practice themselves. As one participant stated, “I thought it was common knowledge to keep yourself healthy and I never termed it self-care. I think this workshop is the first time I heard that actually.” Emphasizing the importance and role of self-care in the sessions was an essential part of the training. Peer facilitators recognized that participating in sexual violence prevention work can be difficult for themselves, as well as many of their peers, so they embraced the idea of talking about self-care prior to the workshops they would deliver. Another participant noted how knowledge of self-care is a benefit for them as an individual even outside of facilitating a sexual violence prevention program.

Participants demonstrated quantitative changes in self-care knowledge. Participant knowledge related to self-care improved significantly between pre-test (19.1 ± 5.8) and post-test (29.3 ± 1.8; *t*(20) = 10.19, *p* < 0.001). These data support the increase in self-care related knowledge reported by participants in the focus group.

Increases in knowledge about components of healthy relationships came up when participants were prompted to discuss what they learned. Participants identified consent and communication as areas of learning during the training. Consent was acknowledged by one participant who stated, “[I] learned a lot about consent, it is very necessary…in relationships you ought to have consent because if you don’t you might be trespassing.” Another participant stated, “in healthy relationships the two…they should have set values on how to relate and you should be able to state your likes and dislikes.” Additionally, participants demonstrated increased self-reported knowledge of healthy relationships. Participant knowledge related to healthy relationships improved significantly between pre-test (22.4 ± 1.6) and post-test (24.0 ± 0.0; *t*(20) = 1.62, *p* < 0.001).

Participants gained a greater understanding of the concepts of coercion and consent. Prior to the training, many participants defined coercion as “forced sex.” After the training, participants demonstrated greater understanding of the nature of coercion, as exemplified by one participant, who responded to an open-ended question that coercion is, “intimidating or expressing threats of immediate or future harm, being it physical [or] emotional, that would force someone to do a sexual activity.” Additionally, a greater understanding of consent was expressed in the post-survey, with individuals recognizing that consent requires a sober, affirmative, verbal answer in the context of a sexual experience. The participants also gained a better understanding of the underlying factors that precipitate sexual violence. This was echoed by a participant who answered on the open-ended question in the post-test that they learned sexual violence is, “gendered in source and targeted around females. So, our gender socialization plays a very critical role in sexual violence.” Further, self-reported sexual violence knowledge increased significantly between pre-test (7.5 ± 1.6) and post-test (9.4 ± 1.5; *t*(20) = 1.95, *p* < 0.001). Sexual health knowledge did not change between pre-test (4.9 ± 0.9) and post-test (4.8 ± 0.8; *t*(20) = –0.05, *p* = 0.771).

#### Attitudes

The participants reflected on their personal attitudes towards sexual harassment, as well as their biases. For instance, in relation to sexual violence one participant said, “In fact I always thought that ladies who wear dresses, very short dresses… and all these things were causes of rape…but now I know that the only cause of rape is the rapist.” Another participant noted that the training helped them think about their personal relationships in relation to sexual violence. This participant noted, “We have never really set out to assess what are our values in relationships and …how to really address them.”

These changes in attitudes about sexual violence were reflected in the modified Rape Myth Acceptance scale. The overall score on the scale increased significantly between pre-test (80.0 ± 15.1) and post-test (93.1 ± 12.2; *t*(20) = 13.13, *p* < 0.001), representing increased rape myth rejection over the course of the training.

Participants also reflected on how their personal biases impacted their understanding of the material included in the primary prevention program, as well as the way they will present the material to others. One participant provided insight on how their biases may impact them as a facilitator: “I found out that in as much as your biases can influence while you are facilitating…” Another participant aptly noted that biases do not have to be a bad thing, stating, “biases are not always negative because we made it feel that [way]…It is all about how to deal with others…knowing that we are bias[ed] in certain areas…and how to deal with it.” Both of these participants highlighted that the participants had not previously had an opportunity to reflect on how their personal biases might impact how they talk to others about sexual violence. All participants reinforced that they valued being able to talk about their biases and think through how this might influence their work as a peer facilitator.

Biases related to gender were also measured quantitatively using the GEM scale. Overall GEM Scale scores increased significantly between pre-test (66.4 ± 4.5) and post-test (68.8 ± 4.5; *t*(20) = 2.37, *p* < 0.001), indicating more equitable attitudes after the training.

#### Learning styles

While learning styles was not a specific evaluation component of this training at the outset, we noted that many of the peer facilitator participants discussed how learning styles impacted their attitudes about the primary prevention program, as well as their thoughts about facilitation. Specifically, the participants discussed engagement, peer-to-peer learning, and training logistics. The first point that the participants noted is that the primary prevention program was engaging because it was interactive and allowed students to work together to discuss the complex topic of sexual violence. As one participant noted, “I also liked how the whole thing was structured with activities because it doesn’t make you bored.” Another participant reflected that the interactive structure distanced the primary prevention program from a traditional lecture when they said, “I think that it’s better that way to involve participants, and also not the boring traditional way of ‘*I am the facilitator so everyone should listen to me*’.”

Second, the participants reiterated how important it was that the primary prevention program was delivered by *peer* facilitators. As one participant noted, “And one thing I have also found interesting and also helpful is also the fact that, facilitation is being done by younger people. So, workshops in Ghana is mostly done by seniors (lecturers), you know, it has to be somebody that is your peer is something that is nice.” Despite the enthusiasm for the structure of the primary prevention program and facilitator training, the participants also noted some concerns that could impact certain types of learners. For instance, some participants expressed that the peer facilitator training was too long and did not occur at a time that worked for all participants. This was noted by one undergraduate peer facilitator who stated, “Am also thinking the next time when there is going to be a training, we will be involving students who are in the system, like learning maybe we can take a time that is suitable so that they will enjoy it.”

#### Skills

A final theme that emerged from the focus group was increased confidence in skills and facilitation techniques. Participants were prompted to explain their level of confidence with facilitating a primary prevention program and, in particular, answering difficult questions. One participant responded that they were “very confident” because they were equipped with “skills and knowledge to facilitate.” Another participant stated that prior to the training they would avoid talking with peers about sexual harassment or violence because they did not know enough. However, after the training, they reporting having the confidence and knowledge to answer their peers’ questions.

Other participants reflected on very practical facilitation skills, such as timing and preparation. Another participant reflected on the importance of preparation prior to facilitation, stating, “You should have enough time to prepare before you actually go out to facilitate. You should be really knowledgeable.” Despite the increased confidence in facilitation skills, a few participants also noted that they wished they had more time to practice delivering the primary prevention program to others.

Participants demonstrated increased self-reported facilitation skills and peer leader skills over the course of training. Self-reported facilitation skills increased significantly from pre-test (21.3 ± 5.9) to post-test (32.5 ± 4.6; *t*(20) = 11.19, *p* < 0.001). Additionally, self-reported peer leader skills increased significantly between pre-test (36.3 ± 6.8) to post-test (41.5 ± 9.6; *t*(20) = 5.17, *p* = 0.008).

### Outcomes by Site

Quantitative outcomes computed for each site (UCC and KNUST) demonstrated that there were significant improvements between pre- and post-training at both universities in the domains of: self-care knowledge, sexual violence knowledge, rape myth acceptance, and facilitation skills (see Table 3 for additional details). The UCC participants also exhibited significant improvements in peer leader skills between pre-test (40.8 ± 5.2) and post-test (46.3 ± 5.2; t(7) = 3.19, p = 0.015). KNUST participants had significant improvements in healthy relationship knowledge between pre-test (21.9 ± 1.8) and post-test (24.0 ± 0.0; t(12) = 4.27, p = 0.001), as well as gender equality between pre-test (65.3 ± 4.9) and post-test (67.9 ± 5.1; t(12) = 3.72, p = 0.003).

### Outcomes by Gender

Table 4 shows quantitative outcomes broken down by gender. Female participants showed significant improvements on all scales except sexual health knowledge between pre- and post-test. Further, male participants significantly improved in their self-care knowledge, healthy relationship knowledge, sexual violence knowledge, rape myth acceptance, and facilitation skills. However, male participants’ scores for the Sexual Health Knowledge scale, Gender Equitable Men scale, and Peer Leader Skills scale did not change between pre- and post-test. Mean scores did not differ for female versus male participants on any of the pre-test measures. However, female participants had significantly greater scores on the following post-tests: Peer Leader Skills (44.4 versus 37.6, t(19) = 1.70, p = 0.009); Facilitation Skills (34.2 versus 30.3, t(19) = 2.04, p = 0.003); Sexual Violence Knowledge (9.9 versus 8.8, t(19) = 1.86, p = 0.001); and Self-Care Knowledge (30.0 versus 28.3, t(19) = 2.33, p < 0.001).

**Table 4:** Paired t-test Comparisons of Pre-test and Post-test Scores by Participant Gender (n = 21).

	Female Participants (n = 12)					Male Participants (n = 9)				

	Pre-Test		Post-Test		*t*-test	Pre-Test		Post-Test		*t*-test
	
	M	SD	M	SD		M	SD	M	SD	

Self-Care Knowledge^1^	19.9	6.7	30.0	0.0	5.18***	18.0	4.5	28.3	2.5	6.46***
Healthy Relationships Knowledge^2^	22.1	1.7	24.0	0.0	3.96**	22.8	1.6	24.0	0.0	2.35*
Sexual Violence Knowledge^3^	7.8	1.7	9.9	0.3	4.29**	7.1	1.5	8.8	2.1	5.77***
Sexual Health Knowledge^4^	4.8	0.8	4.8	0.6	–0.43	4.9	0.9	4.9	0.9	0.00
Modified Rape Myth Acceptance^5^	81.1	14.6	98.9	8.5	4.11**	78.6	16.6	85.4	12.6	3.33*
Gender Equitable Men (GEM) Scale^6^	67.0	3.7	70.2	3.2	4.30**	65.6	5.5	66.9	5.4	1.84
Facilitation Skills^7^	20.8	6.0	34.2	1.6	8.20***	22.0	5.9	30.3	6.3	3.89**
Peer Leader Skills^8^	38.0	6.0	44.4	4.6	3.82**	34.1	7.6	37.6	13.1	1.00

*Note*: M = Mean. SD = Standard Deviation. * *p* < 0.05, ** *p* < 0.01, *** *p* < 0.001. ^1^ Self-Care Knowledge scale (n = 5), scale range 5–30, higher scores representing greater knowledge of self-care practices. ^2^ Healthy Relationship Knowledge (n = 8), scale range 8–24, higher scores represent greater healthy relationship knowledge. ^3^ Sexual Violence Knowledge (n = 2), scale range 2–10, higher scores represent greater sexual violence knowledge. ^4^ Sexual Health Knowledge (n = 6), with 6 representing highest sexual health knowledge. ^5^ Modified Rape Myth Acceptance scale (n = 21), scale range 21–105, higher scores represent greater rape myth rejection. ^6^ Gender Equitable Men (GEM) Scale (n = 24), scale range 24–72, higher scores indicate more equitable gender attitudes. ^7^ Facilitator Skills scale (n = 7), scale range 5–35, higher scores indicate higher self-reported facilitation skills. ^8^ Peer Leader Survey (n = 8), scale range 8–48, higher scores indicate higher self-reported peer leader skills.

## Discussion

Training peer facilitators is a key component of adapting this primary prevention program and represents step seven of the ADAPT-ITT model. This study focused specifically on the TOT model using a Facilitator Training Manual. It resulted in a cadre of 23 trained master trainers at two universities in Ghana who are ready to begin delivering the primary prevention program to their peers. These pieces are critical to the successful implementation and scale-up of the primary prevention program at UCC and KNUST and could be influential in implementation across other universities in Ghana. The manualized nature of the primary prevention program and the facilitator training provides the resources to adapt this program across sub-Saharan Africa. Using primary prevention as a strategy could be the first step in creating change in experiences of sexual violence for university-aged individuals in Ghana.

Sexual violence is a universal problem on university campuses around the world [28], yet there remains a dearth of evidence about available primary prevention programs or training methods for university students in sub-Saharan Africa. There have been limited descriptions of the TOT process or evaluations of this process for sexual violence prevention in sub-Saharan Africa presented in the literature. It appears the TOT model has mainly been described in more broad sexual health and HIV programs in the United States and sub-Saharan Africa [29,30]; with some studies finding that participants liked having peer facilitators better than adult facilitators [31]. Evaluations of peer-led sexual violence prevention training programs in the United States have focused on peer facilitator outcomes after a semester-long course [32], something that is not feasible in most universities or colleges. Other evaluations of peer-led sexual violence prevention training programs have focused on specific groups, such as student attendee outcomes among male fraternity members [33]. Despite this fact, the TOT model is becoming a common practice at universities in the United States to provide mandated primary prevention education. Using rigorous adaptation, evaluation, and training methods provides the evidence-base to allow other schools to utilize this process and model to address sexual violence on their respective campuses.

The participants of the current study found a few parts of the training particularly valuable. The self-care information resonated with the participants from a general wellness perspective. Mental health is often stigmatized and undertreated in sub-Saharan Africa [34], so the participants appreciated thinking about how trauma and vicarious trauma might impact them. They enjoyed brainstorming about ways to take care of themselves while doing work related to sexual violence prevention. The second area of the training the participants highlighted was understanding their own biases. Being able to identify their biases and discuss how that might impact the way they talk to others as a peer facilitator was a useful exercise that led to some vigorous discussion during the training.

Overall, the Ghanaian participants enjoyed learning from the master trainers. The TOT training resulted in significant positive changes in knowledge, attitudes, and skills across a number of domains. While these were individual changes with a small pilot sample, they have the potential to catalyze the community-level changes that affect broader societal attitudes regarding sexual violence. Finally, the UCC master trainers delivered a successful training to their Ghanaian peers at KNUST, showing the efficacy of the TOT model.

### Limitations

The convenience sampling and small sample size of this pilot study may not be representative of the larger student body and highlight the need for replication with a larger sample. However, it was important to pilot this master trainer training with a small group of students due to the time involved. Scale-up of the master training and peer facilitation program will provide the opportunity for a larger scale evaluation. Since the post-test survey data and focus group were conducted immediately after the training, there is potential for social desirability or confirmation bias. Further, since a focus group was conducted at only one location, we do not know if participants at both locations had the same overall takeaways from the training. The sample size for the quantitative analyses was small and although it met all statistical assumptions, results should be replicated with a larger sample size for confirmation. Finally, this study does not examine the relationship between the program and secondary or tertiary outcomes. Despite these limitations, the strengths of this project are the foundation that it is evidence-based, innovative, and culturally adapted. Further, this model was successfully deployed at two different sites and demonstrates that master trainers can successfully train other trainers.

## Conclusion

This manuscript reports on findings from a TOT model utilized to train master trainers for a sexual violence prevention program to be delivered to university students in Ghana. The three-day trainings resulted in significant changes in the in the domains of: self-care knowledge, healthy relationship knowledge, sexual violence knowledge, rape myth acceptance, gender equity, facilitation skills, and peer leadership. The master trainers reflected on how the training appealed to different learning styles. This pilot study begins to establish an evidence base about available primary prevention programs and training methods for students in sub-Saharan Africa. Further research is needed on peer-facilitation, training, and primary prevention programs related to sexual violence for university students in sub-Saharan Africa.
